# Critical signaling pathways in osteoclast differentiation and bone resorption: mechanisms and therapeutic implications for periprosthetic osteolysis

**DOI:** 10.3389/fcell.2025.1639430

**Published:** 2025-08-26

**Authors:** Liangzi Yin, Chenglin Sun, Junjie Zhang, Yan Li, Yansheng Wang, Lunhao Bai, Zeming Lei

**Affiliations:** ^1^ Central Laboratory, Central Hospital Affiliated to Shenyang Medical College, Shenyang, China; ^2^ Laboratory for Hand Bone and Joint Disease, Shenyang Institute of Hand Surgery, Shenyang, China; ^3^ Department of Pathology, Central Hospital Affiliated to Shenyang Medical College, Shenyang, China; ^4^ Department of Hand Surgery, Central Hospital Affiliated to Shenyang Medical College, Shenyang, China; ^5^ Department of Orthopedic Surgery, Shengjing Hospital of China Medical University, Shenyang, China

**Keywords:** periprosthetic osteolysis, bone resorption, signaling pathways, osteoclasts, RANKL/RANK/OPG, NF-κB, MAPK/ERK, therapeutic targets

## Abstract

Bone homeostasis is dynamically regulated by the balance between osteoclast-mediated bone resorption and osteoblast-driven bone formation. Periprosthetic osteolysis (PPO), a major complication following joint arthroplasty, occurs when excessive bone resorption surpasses formation, leading to implant loosening and failure. Emerging evidence highlights the pivotal roles of the RANKL/RANK/OPG axis, nuclear factor-κB (NF-κB) signaling, and mitogen-activated protein kinase/extracellular signal-regulated kinase (MAPK/ERK) cascades in osteoclast differentiation and pathological bone resorption. This review systematically explores the molecular mechanisms by which these pathways regulate osteoclastogenesis and their pathological contributions to PPO. Specifically, we analyze how wear particle-induced inflammation reprograms these signaling networks to exacerbate osteolytic activity. Furthermore, we discuss potential therapeutic strategies targeting these pathways, including pharmacological inhibitors, gene therapy, and dual-target interventions, to restore bone homeostasis. By integrating recent advances in osteoimmunology and translational research, this work provides a comprehensive framework for understanding PPO pathogenesis and developing precision therapies.

## 1 Introduction

Total joint arthroplasty (TJA), including hip and knee replacements, is a cornerstone treatment for end-stage joint diseases, significantly improving patient mobility and quality of life (Bumpass and Nunley, 2012; Mehdipour et al., 2020). However, long-term implant stability remains a critical challenge, with PPO being a leading cause of aseptic loosening and revision surgeries (Pajarinen et al., 2014). As the global demand for TJA rises alongside increasing life expectancy, PPO has emerged as a major clinical burden, compromising surgical outcomes and escalating healthcare costs. PPO is primarily driven by chronic inflammatory responses to wear particles (e.g., polyethylene, metal, or ceramic debris) generated at the implant-bone interface (Tao et al., 2023; Longhofer et al., 2017; Sukur et al., 2016). These particles are phagocytosed by macrophages, triggering the excessive release of pro-inflammatory cytokines such as tumor necrosis factor-α (TNF-α), interleukin-1 (IL-1) and interleukin-6 (IL-6). These cytokines orchestrate a pathological cascade that promotes osteoclast differentiation, hyperactivation, and bone resorption, while simultaneously suppressing osteoblast-mediated bone formation (Weivoda and Bradley, 2023; Zhang L. et al., 2020; Yin et al., 2023). Osteoclasts, the principal bone-resorbing cells, undergo a tightly regulated differentiation process governed by intricate signaling networks. Among these, the RANKL/RANK/OPG axis, NF-κB signaling, and MAPK/ERK pathways have been identified as central regulators of osteoclastogenesis and bone metabolism (Boyle et al., 2003). Recent advancements in molecular biology and genetic engineering have deepened our understanding of these pathways in PPO pathogenesis. For instance, aberrant activation of NF-κB, and MAPK/ERK signaling has been linked to inflammatory bone loss in osteoporosis, rheumatoid arthritis, and PPO (Hu et al., 2020; Zhou et al., 2024; Gan et al., 2024). Preclinical studies targeting these pathways via pharmacological inhibitors, monoclonal antibodies, or gene-editing approaches have shown promise in mitigating osteolysis (Liu and Maeyama, 2016; Adriaansen et al., 2006; Nakajima, 2006; Wei et al., 2018). Nevertheless, critical knowledge gaps persist regarding the dynamic interplay between these pathways, their spatiotemporal regulatory mechanisms, and the long-term efficacy and safety of pharmacological interventions. Furthermore, developing multi-targeted combinatorial strategies to enhance therapeutic outcomes while minimizing adverse effects represents a pivotal direction for future research (Novack, 2011; Mehta et al., 2022).

This review systematically examines the molecular mechanisms by which three canonical signaling pathways (RANKL/RANK/OPG, NF-κB and MAPK/ERK) regulate osteoclast differentiation and bone resorption. We particularly focus on their therapeutic implications for periprosthetic osteolysis, aiming to provide a comprehensive theoretical framework and actionable directions for elucidating pathological mechanisms and optimizing clinical interventions.

## 2 Pathogenesis of periprosthetic osteolysis

PPO is fundamentally driven by a self-perpetuating cycle of chronic inflammation and pathological bone remodeling, triggered by the interplay between wear particle-mediated immune activation and dysregulated cytokine signaling. At the core of this process lies the aberrant activation of osteoclasts, which overwhelms osteoblast-mediated bone formation, culminating in progressive bone loss and implant destabilization.

### 2.1 Inflammatory response induced by bioactive wear particles

PPO is a predominant complication following TJA, primarily driven by chronic inflammation triggered by bioactive wear particles generated at the implant-bone interface (Harris, 2001; Panez-Toro et al., 2023; Connors et al., 2022). These particles, including polyethylene (PE), metal debris, ceramic fragments, and bone cement residues are released due to mechanical wear and corrosion of prosthetic components over time. Particle characteristics such as size (0.1–10 µm), morphology (sharp-edged vs. spherical), and chemical composition critically influence their bioreactivity and inflammatory potential (Devane et al., 1995; Morawietz and Krenn, 2014). For instance, submicron-sized particles and elongated shapes exhibit stronger pro-inflammatory effects compared to larger or spherical particles, while metallic debris may provoke delayed hypersensitivity reactions (Bitar and Parvizi, 2015; Atkins et al., 2011; Nine et al., 2014; Wang et al., 2004). Upon infiltration into periprosthetic tissues, wear particles are phagocytosed by macrophages, which are key mediators of the innate immune response (Nich et al., 2013). Particle phagocytosis activates pattern recognition receptors (e.g., Toll-like receptors, TLRs) and inflammasomes, leading to the overproduction of pro-inflammatory cytokines (e.g., TNF-α, IL-1β, IL-6) and chemokines (e.g., MCP-1, MIP-1α) (Xie et al., 2023). These soluble factors collectively establish a pro-osteolytic microenvironment through multifaceted mechanisms. TNF-α and IL-1β synergistically upregulate receptor activator of nuclear factor-κB ligand (RANKL) expression in osteoblasts and fibroblasts, thereby accelerating osteoclast differentiation and bone resorption through paracrine signaling (Luo et al., 2018; Xu et al., 2023; Wei et al., 2005). Concurrently, chronic inflammation disrupts osteoblast-mediated bone formation by suppressing Wnt/β-catenin signaling pathways and inducing apoptotic cascades, which impair osteoblast proliferation and matrix deposition (Hu et al., 2024; Shahnazari et al., 2008; Ma and Hottiger, 2016). Furthermore, chemokine-mediated recruitment of monocytes, lymphocytes, and macrophages, particularly through monocyte chemoattractant protein-1 (MCP-1), perpetuates inflammatory cell infiltration at the implant site, establishing a self-sustaining cycle of tissue damage (Deshmane et al., 2009; Gschwandtner et al., 2019). Critically, the dynamic polarization of macrophages underlies the pathological progression of PPO. Pro-inflammatory M1 macrophages dominate the early-phase inflammatory milieu, driving osteoclast activation through cytokine secretion. In contrast, The M2 phenotype is primarily observed in macrophages under non-inflammatory conditions, where it promotes tissue repair and homeostasis (Gao X. R. et al., 2018; Jia et al., 2024; Pajarinen et al., 2021). However, premature or excessive polarization towards the M2 phenotype during the early stages impairs healing and can lead to fibrous encapsulation or fibrous tissue formation instead of bone regeneration (He et al., 2022). Thus, the polarization of macrophages (M1/M2) itself is important for the determination of osteoclastogenesis. Preclinical validation from animal models confirms that localized particle exposure exacerbates osteolysis through TLR/NF-κB and MAPK pathway activation, highlighting the centrality of inflammatory signaling in disease pathogenesis (Ren et al., 2004; Philbrick et al., 2018; Jiang et al., 2018).

### 2.2 Role of cytokines

PPO is characterized by sterile inflammatory bone destruction, wherein chronic low-grade inflammation plays a pivotal role in driving pathological bone resorption (Gallo et al., 2013a; Gallo et al., 2013b; Anderson et al., 2008). Inflammation, as a protective response to foreign particles, tissue damage, or mechanical stress, involves complex interactions among immune cells, cytokines, and signaling pathways (Wang and Medzhitov, 2019; Yeung et al., 2018; Zindel and Kubes, 2020; Gong et al., 2020). Among these, cytokines, which are soluble proteins secreted by macrophages, monocytes, and fibroblasts, act as key mediators of immune and inflammatory responses. Macrophages, the primary phagocytes of wear particles, are central to PPO pathogenesis. Their abundance in periprosthetic tissues correlates positively with the severity of inflammation and osteolysis (Haringman et al., 2005). Macrophages exhibit remarkable plasticity, differentiating into distinct phenotypes depending on the microenvironment. Classically activated M1 macrophages promote inflammation and bone resorption, whereas alternatively activated M2 macrophages exert anti-inflammatory and tissue-repairing effects (Chen et al., 2023; Sica and Mantovani, 2012). *In vitro* studies demonstrate that macrophages phagocytosing polymethylmethacrylate (PMMA) particles predominantly polarize toward the M1 phenotype, releasing pro-inflammatory cytokines such as TNF-α, IL-1, IL-6, prostaglandin E2 (PGE2) and nitric oxide (NO) (Chen T. H. et al., 2019; Tsutsumi et al., 2009; Udagawa et al., 2021; Ruaro et al., 2018). These cytokines synergistically recruit inflammatory cells (e.g., Th1 lymphocytes) and activate osteoclasts, thereby amplifying bone resorption (Comerford et al., 2014; White et al., 2013). Concurrently, M1 macrophages secrete chemokines (e.g., MCP-1; macrophage inflammatory protein-1α, MIP-1α) and proteolytic enzymes (e.g., matrix metalloproteinases, MMPs), which facilitate immune cell infiltration and extracellular matrix degradation (Gao X. et al., 2018; Jämsen et al., 2017; Wang W. et al., 2022; Krzyszczyk et al., 2018). M2 macrophages polarized by IL-4/IL-13 contribute to tissue repair and inflammation resolution via anti-inflammatory cytokines (e.g., IL-10, TGF-β). While they indirectly suppress osteoclastogenesis by stimulating OPG production in osteoblasts (Wang W. et al., 2022; Souza and Lerner, 2013; Sun et al., 2021), their role in bone formation is permissive rather than inductive. Robust osteoinduction requires direct anabolic signals such as BMP-2 or Wnt activation, independent of macrophage-mediated priming (Pederson et al., 2008; Urist, 1965; Teti, 2013).

Osteocytes, the predominant cells within bone tissue, play a pivotal role in the pathogenesis of PPO. Acting as mechanosensors, they detect wear particles and alterations in the mechanical environment via their dendritic network (Metzger and Narayanan, 2019; Panez-Toro et al., 2023). The disruption of this network by wear particles triggers osteocytes to release extracellular vesicles (EVs) containing factors such as RANKL, sclerostin, and IL-6, along with soluble signaling molecules. These released mediators act upon osteoclast precursors and bone-lining cells, potently stimulating osteoclastogenesis, osteoclast activation, and inflammatory responses, while simultaneously inhibiting bone formation (Harada et al., 2021). This cascade culminates in a self-perpetuating vicious cycle, ultimately driving progressive periprosthetic bone destruction.

### 2.3 Synergistic effects of cytokines in bone resorption

The inflammatory microenvironment in PPO is orchestrated by a network of pro-osteolytic cytokines that synergistically drive pathological bone remodeling. Among these mediators, TNF-α serves as a central regulator, promoting osteoclast differentiation and bone resorption through both RANKL-dependent and RANKL-independent mechanisms. TNF-α further amplifies osteolytic activity by stimulating osteoblasts to secrete IL-6 and PGE2, creating a feedforward loop of inflammation (Kobayashi et al., 2000; Millet et al., 1998; Fuller et al., 2002). Clinically, elevated TNF-α levels are consistently observed in periprosthetic tissues of PPO patients, with *in vitro* studies demonstrating that titanium particles synergize with TNF-α to enhance osteoclastogenesis, underscoring the interplay between wear debris and cytokine signaling (Ge et al., 2018; Schwarz et al., 2000). Another critical contributor, IL-1β not only directly induces osteoclast differentiation but also activates fibroblasts and synovial cells to release MMPs and PGE2 (Gowen et al., 1983). Experimental evidences from murine calvarial cultures have revealed that IL-1β upregulates MMP-2, MMP-3, MMP-9, and MMP-13 expression, accelerating bone matrix degradation (Kusano et al., 1998; Cheng et al., 2020; Ruscitti et al., 2015). Additionally, IL-6 predominantly produced by osteoblasts under inflammatory conditions, exacerbates bone catabolism by modulating the RANKL/OPG balance and enhancing osteoclast precursor sensitivity to RANKL. This indirect pathway positions IL-6 as a key mediator linking inflammation to imbalanced bone remodeling (Feng et al., 2017; McGregor et al., 2019). IL-10 significantly inhibits collagen degradation and maintains extracellular matrix homeostasis by stimulating TIMP-1 expression while suppressing MMPs (such as MMP-9 and MMP-13), particularly in foreign body reactions and inflammatory environments (Ye et al., 2011; Lacraz et al., 1995). Osteoblasts also can express MMP-2, MMP-8 and TIMP-1/-2/-3, and participate in bone matrix remodeling by degrading ECM molecules (Hatori et al., 2004). IL-17 inhibits bone formation by up-regulating SOST/sclerostin and suppressing the Wnt/β-catenin signaling pathway. Meanwhile, through the increase of RANKL mediated by SOST and the activation of Cathepsin K, bone resorption is promoted. The activation of the Wnt/β-catenin pathway (such as the blocking of the SOST gene) is the key to promoting bone formation and combating bone loss (Jiao et al., 2023; Zhang Z. H. et al., 2020).

The involvement of PGE2 in inflammation-mediated bone resorption remains debated. While some studies emphasize the critical role of PGE2 in osteolysis, particularly through its synergistic interaction with TNF-α and IL-1β to amplify osteoclast activity, other studies indicate that dialysis-mediated depletion of PGE2 does not attenuate bone resorption in specific experimental models (Lader and Flanagan, 1998; Miyaura et al., 2003). This discrepancy may stem from context-dependent effects of PGE2, potentially mediated through interactions with other inflammatory mediators and tissue-specific signaling networks (Sheibanie et al., 2007). Chemokines such as MCP-1 and MIP-1α are pivotal in recruiting monocytes, macrophages, and lymphocytes to periprosthetic sites, perpetuating the inflammatory cascade. Their overexpression in PPO tissues correlates with enhanced osteoclast recruitment and bone loss. Notably, IFN-γ exhibits dual effects in bone remodeling. On one hand, it inhibits osteoclastogenesis by promoting TRAF6 degradation in osteoclast precursors; on the other, it enhances antigen presentation and MHC class II expression, which may indirectly stimulate pro-inflammatory responses in periprosthetic tissues (Takayanagi et al., 2000; Niu et al., 2022). These findings collectively outline the roles of pro-inflammatory cytokines in PPO. A summarized comparison of key cytokines, their sources, signaling pathways, and pathological effects is presented in Table 1.

**TABLE 1:** Key cytokines involved in periprosthetic osteolysis and their pathogenic roles.

Cytokine	Primary cellular source	Signaling target/Pathway	Pathological role in PPO	Key references
TNF-α	M1 macrophages, T cells, fibroblasts	TNFR1 → NF-κB, MAPK	Induces RANKL expression, directly promotes osteoclastogenesis, and stimulates IL-6/PGE2	Ge et al. (2018), Schwarz et al. (2000)
IL-1β	Macrophages, synovial cells	IL-1R → NF-κB, Wnt/β-catenin	Activates MMPs and PGE2, enhances TNF-α signaling, facilitates matrix degradation	Kusano et al. (1998), Yu and Canalis (2020)
IL-6	Osteoblasts, macrophages	IL-6R/gp130 → JAK/STAT3	Increases RANKL/OPG imbalance, sensitizes osteoclast precursors to RANKL	Feng et al. (2017), McGregor et al. (2019)
IL-10	M2 macrophages, Treg cells	IL-10R → STAT3	Suppresses MMPs, stimulates OPG production, promotes resolution of inflammation	Souza and Lerner (2013), Ye et al. (2011)
IL-17	Th17 cells	IL-17R → NF-κB, MAPK	Promotes sclerostin expression, enhances RANKL and Cathepsin K activity	Jiao et al. (2023), Zhang et al. (2020b)
MCP-1	Macrophages, fibroblasts	CCR2-mediated chemotaxis	Recruits inflammatory cells (monocytes/macrophages) and maintains inflammatory microenvironment	Deshmane et al. (2009), Gschwandtner et al. (2019)
MIP-1α	M1 macrophages, osteoblasts	CCR1/CCR5 pathway	Promotes osteoclast precursor recruitment and enhances resorptive activity	Gao et al. (2018a), Jämsen et al. (2017), Wang et al. (2022c)
IFN-γ	NK cells, Th1 cells	TRAF6-dependent, MHC pathways	Inhibits osteoclastogenesis via TRAF6 degradation but promotes antigen presentation	Niu et al. (2022), Takayanagi et al. (2000)
PGE2	Fibroblasts, macrophages	EP receptors → cAMP/PKA	Enhances osteoclastogenesis in synergy with TNF-α and IL-1β	Lader and Flanagan (1998), Sheibanie et al. (2007)

Given the central role of chronic inflammation and osteoclast overactivation in the pathogenesis of PPO, elucidating the molecular signaling pathways that govern these processes is essential for identifying therapeutic targets. Among the complex regulatory networks, the RANKL/RANK/OPG axis, NF-κB signaling, and MAPK/ERK pathways serve as key mediators of osteoclast differentiation, survival, and bone resorption. These pathways not only respond to inflammatory cues but also integrate mechanical and hormonal signals, rendering them crucial nodes in the progression of PPO. Subsequently, we dissect the structural and functional characteristics of these canonical pathways and explore how their dysregulation contributes to pathological bone loss. Moreover, we discuss emerging therapeutic interventions that modulate these pathways to restore bone homeostasis and prevent aseptic loosening and subsequent implant failure.

## 3 RANKL/RANK/OPG signaling pathway

As the core hub for bone homeostasis regulation, the RANKL/RANK/OPG signaling axis plays a decisive role in pathological and physiological bone remodeling by dynamically balancing osteoclast differentiation and bone resorption. To further elucidate its molecular mechanisms, the following sections will systematically dissect the composition, function, and multi-level regulatory network of this pathway.

### 3.1 Structural composition of RANKL/RANK/OPG

During the late 1990s, the RANKL/RANK/OPG system was elucidated as a pivotal regulator of bone remodeling (Lacey et al., 1998; Yasuda et al., 1998). The RANKL/RANK/OPG signaling axis belongs to the tumor necrosis factor (TNF) receptor-ligand superfamily. RANKL, encoded by the TNFSF11 gene on human chromosome 13, is a type II transmembrane protein that forms homotrimers (Walsh and Choi, 2014). Its extracellular domain can be cleaved by MMPs to generate soluble RANKL (sRANKL), which retains biological activity (Li et al., 2022; Takegahara et al., 2022). Two receptors have been reported for RANKL, the membrane-bound receptor RANK, also known as TNF-related activation-induced cytokine receptor or TNF receptor superfamily member 11A (TNFRSF11A), and the soluble decoy receptor OPG or TNFRSF11B. RANK (TNFRSF11A), located on chromosome 18, is a transmembrane receptor expressed on osteoclast precursors, mature osteoclasts, and immune cells. Upon binding to RANKL, RANK recruits adaptor proteins such as TRAF6 to initiate downstream signaling cascades (Walsh and Choi, 2014; Liu and Zhang, 2015). Osteoprotegerin (OPG), encoded by the TNFRSF11B gene on chromosome 8, is a soluble decoy receptor that competitively inhibits RANKL-RANK interaction by binding RANKL with higher affinity than RANK itself (Liu and Zhang, 2015). Structural studies reveal that OPG exists as a dimer, while RANKL and RANK function as trimers and monomers, respectively (Liu et al., 2010). OPG dimerizes before binding to RANKL (Munasinghe et al., 2017). Moreover, the OPG-membrane RANKL leads to the internalization of OPG mediated by RANKL via the clathrin pathway to the lysosomal and proteasomal degradation (Tat et al., 2006).

### 3.2 RANKL/RANK/OPG and bone resorption

**FIGURE 1:**
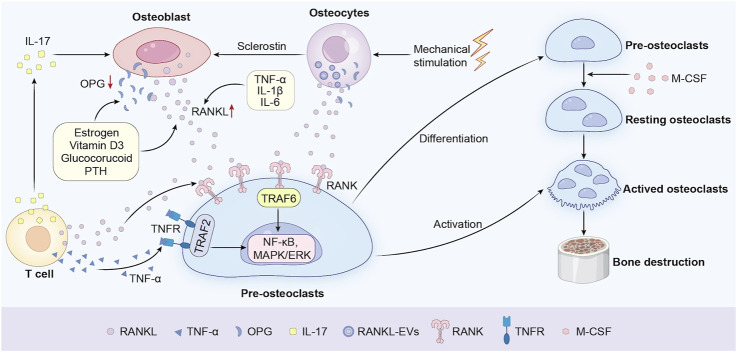
Molecular mechanisms of the RANKL/RANK/OPG axis in osteoclastogenesis.

Bone homeostasis is meticulously regulated by the dynamic equilibrium between osteoclast-mediated bone resorption and osteoblast-driven bone formation. Central to this balance is the RANKL/RANK/OPG signaling axis, a critical regulatory system governing osteoclast differentiation and activity (Figure 1). RANKL, a transmembrane protein predominantly secreted by osteoblasts and activated T cells, serves as the master cytokine for osteoclastogenesis. Additionally, vascular endothelial cells contribute to osteoclastogenesis by secreting RANKL, particularly in inflammatory bone microenvironments (O'Brien, 2010). Osteocytes, in both physiological and pathological conditions, have also emerged as a major source of RANKL. Studies have shown that osteocytes play a key role in the formation of osteoclasts during bone remodeling in adult mice. For instance, Xiong et al. found through conditional knockout of the RANKL gene that the loss of RANKL in osteocytes would lead to osteopetrosis, indicating that osteocytes mediate bone resorption through RANKL in bone remodeling (Xiong et al., 2015). Notably, groundbreaking research by Nakashima et al. demonstrated that mice with osteocyte-specific deletion of RANKL exhibit severe osteopetrosis, resulting from impaired osteoclast formation, thereby highlighting the essential role of osteocytes in bone resorption (Nakashima et al., 2011). Upon binding to its receptor RANK on osteoclast precursors, RANKL initiates a cascade of intracellular events, including the recruitment of Tumor Necrosis Factor Receptor-Associated Factor 6 (TRAF6). This adaptor protein activates downstream signaling pathways such as NF-κB, MAPK/ERK, and Nuclear Factor of Activated T-cells 1 (NFATc1), which collectively drive osteoclast differentiation, maturation, and bone-resorbing activity (Park et al., 2017; Tan et al., 2017; Gohda et al., 2005). Besides, the M-CSF/CSF-1R pathway can facilitate osteoclast precursor growth and mature osteoclast survival. Meanwhile, the RANKL/RANK pathway acts as an essential choreographer to gracefully orchestrate the dance of osteoclast differentiation and maturation (Veis and O'Brien, 2023). The indispensable role of RANKL in osteoclast biology is underscored by genetic studies. RANKL-deficient mice exhibit severe osteopetrosis, a condition characterized by abnormally dense bone resulting from the absence of functional osteoclasts (Bucay et al., 1998; Yasuda, 2021; Lo Iacono et al., 2012). Conversely, OPG, a soluble decoy receptor that competitively inhibits RANKL-RANK interaction, acts as a natural brake on osteoclast activity. The balance of the OPG and RANKL expression is vital in bone metabolism and homeostasis. OPG-knockout mice develop early-onset osteoporosis, marked by excessive bone resorption and fragility fractures, highlighting the delicate balance between RANKL and OPG in maintaining skeletal integrity (Min et al., 2000; Bucay et al., 1998). Clinically, dysregulation of the RANKL/OPG ratio is implicated in bone metabolic disorders such as osteoporosis, rheumatoid arthritis, and PPO (Jura-Półtorak et al., 2021). For example, elevated RANKL expression in periprosthetic tissues correlates with accelerated osteolysis and implant failure (Veigl et al., 2007). In the context of PPO, a leading cause of implant failure following joint arthroplasty, the RANKL/OPG axis is hijacked by inflammatory mediators. Wear particles (e.g., polyethylene debris) at the implant-bone interface induce chronic inflammation, triggering macrophages and fibroblasts to secrete pro-osteolytic cytokines such as TNF-α and IL-1β. These cytokines synergistically upregulate RANKL expression while suppressing OPG production, creating a microenvironment conducive to osteoclast hyperactivation (Panez-Toro et al., 2023). Clinical studies corroborate this mechanism, demonstrating that periprosthetic tissues from patients with implant loosening exhibit significantly elevated RANKL levels and reduced OPG, correlating with accelerated osteolysis and radiographic evidence of bone loss (Veigl et al., 2007; Crotti et al., 2004). The regulatory proteins RANKL, OPG, and sclerostin can also be released into the extracellular medium by the osteocytes within extracellular vesicles (EVs) via a Ca^2+^-dependent mechanotransduction signaling pathway that induces changes in the cytoskeletal arrangement (Morrell et al., 2018). However, the main mechanism is not fully clear because the osteocytes are embedded in the bone matrices while the osteoclast precursors are localized in the bone marrow cavities. Notably, therapeutic strategies targeting this axis, such as Denosumab (a monoclonal antibody against RANKL), have shown promise in reducing osteoclast activity in PPO models, though challenges remain in achieving localized delivery without systemic side effects (Sköldenberg et al., 2016).

Recent advances in single-cell RNA sequencing have unveiled spatial heterogeneity in RANKL expression within the bone microenvironment, suggesting that osteoblasts and immune cells may contribute differentially to pathological osteolysis (Yu et al., 2021; Tsukasaki and Takayanagi, 2022). Furthermore, novel bispecific antibodies simultaneously targeting RANKL and inflammatory cytokines (e.g., TNF-α/IL-6) are under investigation to disrupt the feedforward loop of inflammation and bone resorption (Hashizume et al., 2008). These developments underscore the potential of multi-target therapies to restore RANKL/OPG equilibrium and mitigate PPO progression.

### 3.3 Regulation of the RANKL/RANK/OPG pathway

The RANKL/RANK/OPG signaling axis, serving as the core regulatory mechanism for bone metabolism, is dynamically and synergistically modulated by hormonal, mechanical, and inflammatory cues.

#### 3.3.1 Hormonal regulation

Estrogen deficiency contributes to osteoporosis by upregulating RANKL expression and downregulating OPG expression. This mechanism is particularly evident in postmenopausal women, as the decline in estrogen levels leads to an imbalance in bone remodeling, increased bone resorption and reduced bone formation (Khosla and Riggs, 2005; Eghbali-Fatourechi et al., 2003). Streicher et al. (2017) demonstrated through bone marrow transplantation models and chimeric mouse experiments that estrogen directly suppressed RANKL transcription in bone lining cells via ERα, thereby inhibiting osteoclast activity. RANKL forms an “*in situ* signaling pool” on the bone surface, regulating the differentiation of adjacent osteoclast precursors. Glucocorticoids (GC) promote the differentiation and activation of osteoclasts by inhibiting the expression of OPG in osteoblasts and stimulating the expression of RANKL. For instance, Hofbauer et al.’s research demonstrated that dexamethasone can significantly inhibit the mRNA and protein expression of OPG in human osteoblasts while increasing the expression of RANKL (Hofbauer et al., 2001). This effect of reduced OPG and increased RANKL leads to enhanced bone resorption, thereby triggering osteoporosis (Piemontese et al., 2016; Hofbauer et al., 1999). Specifically, prolonged prednisolone exposure downregulated GR expression (1.26-fold reduction in bone marrow cells) and disturbed the OPG/RANKL ratio (2-fold decrease), leading to NF-κB pathway activation (1.48-fold increase in nuclear p65 phosphorylation) and subsequent osteoclast precursor proliferation. These effects are paralleled by impairments in the vitamin D autocrine or paracrine system, characterized by reduced CYP27B1 and vitamin D receptor (VDR) levels (Shymanskyi et al., 2018). Remarkably, cholecalciferol supplementation restores GR-dependent signaling, inhibits RANKL/NF-κB crosstalk via VDR activation, and rebalances the OPG/RANKL ratio, thereby mitigating osteoclastogenesis. These findings highlight vitamin D3 as a potential adjuvant therapy for rescuing GC-induced dysregulation of RANKL-OPG-GR networks (Shymanskyi et al., 2018). Emerging evidence delineates a biphasic regulatory paradigm of parathyroid hormone (PTH) on skeletal homeostasis through dynamic regulation of the RANKL/OPG axis (Saini et al., 2013). Chronic PTH exposure induces osteoclastogenesis via NF-κB pathway activation, achieved through cAMP/PKA-mediated transcriptional reprogramming in osteoblasts/osteocytes that elevates RANKL expression (1.5-fold RANKL/OPG mRNA ratio) while suppressing OPG production, with synergistic amplification via MCP-1-dependent osteoclast precursor chemotaxis (Wein and Kronenberg, 2018). In contrast, intermittent PTH administration promotes bone anabolism by suppressing sclerostin to enhance Wnt/β-catenin signaling, while restoring RANKL/OPG equilibrium through EphrinB2-EphB4-mediated bidirectional osteoblast-osteoclast communication (Silva and Bilezikian, 2015). This dual action positions PTH as a context-dependent modulator of bone turnover, orchestrating catabolic-anabolic balance through crosstalk between RANKL/OPG dynamics and osteocyte-derived Wnt inhibitors (Silva and Bilezikian, 2015).

#### 3.3.2 Inflammatory cytokine-mediated modulation

Pro-inflammatory cytokines such as TNF-α, IL-1β, and IL-6 amplify RANKL production in osteoblasts and synovial fibroblasts, exacerbating osteoclast activation in inflammatory bone diseases (Ruscitti et al., 2015; Hashizume et al., 2008; Jones et al., 2011). The study by Hashizume et al. (2008) revealed that in the synovium of rheumatoid arthritis, IL-6 trans-signaling activates the JAK/STAT3 pathway through the IL-6/sIL-6R complex, directly upregulating RANKL expression. TNF-α and IL-17 synergistically enhance this effect by paracrine secretion of IL-6, while IL-1β activates RANKL through an independent pathway. Experiments have demonstrated that the IL-6-RANKL axis drives osteoclast differentiation via NFATc1 upregulation, and tocilizumab (an IL-6R inhibitor) can specifically block this pathway (Uciechowski and Dempke, 2020). These findings suggest that targeting trans-signaling is an effective strategy for inhibiting inflammatory bone destruction. While IL-6 trans-signaling drives osteoclastogenesis *in vitro*, its role in PPO is corroborated by elevated IL-6/sIL-6R levels in periprosthetic synovial fluids (Singh et al., 2021; Kotake et al., 1996). However, clinical translation faces challenges: Redundant cytokine networks may limit efficacy of single-target inhibitors (e.g., tocilizumab reduces RA bone erosion but impairs fracture healing) (Le Goff et al., 2010). Localized delivery strategies show promise in preclinical PPO models. IL-1β can directly enhance the expression of RANKL in osteoblasts and promote osteoclast differentiation by activating signaling pathways such as NF-κB and Wnt/β-catenin (Yu and Canalis, 2020; Kohli and Kohli, 2011). Studies have shown that TNF-α-induced RANKL expression partially depends on the mediation of IL-1β. For example, TNF-α upregulates RANKL by stimulating bone marrow stromal cells to secrete IL-1 (Wei et al., 2005). IL-1β can also synergize with TNF-α to further amplify osteoclast activation (Carmona-Rivera et al., 2024).

#### 3.3.3 Pharmacological interventions

Denosumab, a monoclonal antibody targeting RANKL, is clinically approved for osteoporosis and cancer-related bone loss. A recent study by El-Masri et al. (2024) has demonstrated that bone surface cells play a central role in bone metabolism through dynamic regulation of the RANKL/OPG balance. Utilizing *in situ* hybridization and single-cell RNA sequencing, the study revealed that following discontinuation of denosumab (a RANKL inhibitor), RANKL mRNA expression in trabecular bone surface cells was significantly upregulated (+40%, P < 0.01), while OPG mRNA levels decreased by 30%, resulting in an imbalanced RANKL/OPG ratio (El-Masri et al., 2024). Besides, Clinical data show that 6 months after discontinuing denosumab, the serum RANKL level in patients significantly increased, followed by an increase in the osteoclast activity marker TRAcP5b. This indicates that the accumulation of RANKL directly promotes an increase in the number of osteoclasts and enhanced bone resorption (Sølling et al., 2023). These findings elucidate the cellular mechanism underlying the high incidence of rebound bone resorption in trabecular-rich regions such as vertebral bodies, suggesting that targeting signaling pathways in bone surface cells may optimize anti-osteoporotic therapeutic strategies.

Natural compounds (e.g., genistein) and bisphosphonates indirectly modulate the pathway by altering the RANKL/OPG balance (Zhu et al., 2020; Hooshiar et al., 2022). Hooshiar et al. (2022) systematically elucidate the pleiotropic pharmacological mechanisms by which soy isoflavones (genistein, daidzein) modulate the RANK/RANKL/OPG axis to restore bone metabolic homeostasis. Mechanistic studies revealed these phytoestrogens concurrently suppress RANKL expression and enhance OPG production, thereby normalizing the RANKL/OPG ratio through dual nuclear receptor engagement: ERβ-mediated transcriptional repression of RANKL promoters and PPARγ-dependent NF-κB pathway inhibition (Viereck et al., 2002; García Palacios et al., 2005). Bisphosphonates inhibit farnesyl pyrophosphate synthase (FPPS) in the mevalonate pathway, reduce the expression of RANKL, and increase the expression of OPG, thereby lowering the RANKL/OPG ratio and inhibiting osteoclast activity (de Castro et al., 2019; Tsubaki et al., 2012).

#### 3.3.4 Mechanical stress

Postoperative reduction in mechanical loading following arthroplasty (e.g., due to pain or immobilization) precipitates disuse osteoporosis, accelerating PPO through osteocyte-mediated dysregulation of bone remodeling. Mechanistically, Osteocytes are the primary mechanosensitive cells in bone, capable of sensing changes in mechanical loading (Bonewald, 2011). When mechanical loading decreases (such as during prolonged bed restgr or in a microavity environment), osteocytes downregulate bone formation inhibitors like sclerostin and simultaneously upregulate the expression of RANKL significantly (Mancuso et al., 2022). Sclerostin inhibits osteoblast activity by antagonizing the Wnt/β-catenin signaling pathway, while promoting RANKL-mediated bone resorption (Lin et al., 2009). Clinically, reduced loading after arthroplasty (e.g., due to pain or immobilization) mimics disuse osteoporosis, accelerating PPO via osteocyte-mediated RANKL overexpression. For example, the hindlimb unloading (HLU) experiment showed that osteocyte apoptosis increased by 3–4 times within 5 days, while RANKL expression was upregulated, directly activating osteoclast differentiation (Cabahug-Zuckerman et al., 2016). Besides, A meta-analysis reported 2.1-fold higher osteolysis risk in patients with limited postoperative weight-bearing, underscoring the need for early mechanotherapeutic interventions (Smeeing et al., 2015). Reduced mechanical loading upregulates RANKL in osteocytes, linking disuse osteoporosis to enhanced osteoclast activity (Buck and Stains, 2024; Cabahug-Zuckerman et al., 2016, Matsushita et al., 2020). Emerging work by Buck and Stains (2024) delineated a mechanotransduction cascade through which mechanical loading suppresses osteoclastogenesis via osteocytic regulation of RANKL/OPG dynamics. A recent study indicates that osteoclasts may also directly respond to mechanical stimulation, but how this response may participate in anabolic processes is not settled (Dsouza and Komarova, 2024).

Post-arthroplasty disuse osteoporosis is orchestrated by osteocyte-driven RANKL overexpression, necessitating timely, load-dependent mechanotherapy to mitigate PPO risk. Individualized rehabilitation protocols balancing fixation stability and early dynamic loading should be prioritized.

## 4 NF-κB signaling pathway

The NF-κB signaling pathway serves as a molecular bridge between inflammation and bone resorption, and its aberrant activation is the core mechanism underlying particle-induced osteolysis. To gain a deeper understanding of its pathological contributions, this section first focuses on the structural characteristics of the NF-κB family and its activation mechanisms.

### 4.1 Structural composition of NF-κB signaling

The NF-κB family comprises a group of evolutionarily conserved transcription factors that regulate diverse biological processes, including inflammation, immune responses, and osteoclast activation. In mammals, the NF-κB family includes five members: RelA (p65), RelB, c-Rel, p50 (NF-κB1), and p52 (NF-κB2). These proteins share a conserved Rel homology domain (RHD) at their N-terminus, which mediates dimerization, nuclear localization, and DNA binding. RelA, RelB, and c-Rel contain a transactivation domain (TAD) for transcriptional activation, whereas p50 and p52, processed from precursors p105 and p100, respectively, lack TAD and primarily act as transcriptional repressors in their homodimeric forms (Baeuerle and Henkel, 1994; Thanos and Maniatis, 1995; Baldwin, 1996). NF-κB activation occurs through two distinct pathways. The canonical pathway is initiated by pro-inflammatory cytokines such as TNF-α and IL-1β or pathogen-associated molecular patterns (PAMPs) engaging receptors including TNFR, IL-1R, and TLRs. Ligand binding recruits adaptor proteins (e.g., TRADD, MyD88), leading to activation of the IκB kinase (IKK) complex (IKKα/IKKβ/NEMO). IKKβ phosphorylates IκBα, marking it for proteasomal degradation. This releases NF-κB dimers (e.g., RelA/p50), which translocate to the nucleus to induce pro-inflammatory cytokine and anti-apoptotic gene expression (Lawrence, 2009; Guo et al., 2024). In contrast, the non-canonical pathway is activated by receptors such as CD40 and B-cell activating factor receptors (BAFF-R). This cascade depends on NF-κB-inducing kinase (NIK)-mediated phosphorylation of IKKα, which catalyzes the proteolytic processing of p100 to p52. The resulting transcriptionally active RelB/p52 dimers regulate genes involved in lymphoid organ development and chronic inflammatory responses (Cildir et al., 2016; Gao et al., 2021).

### 4.2 NF-κB signaling and bone resorption

**FIGURE 2:**
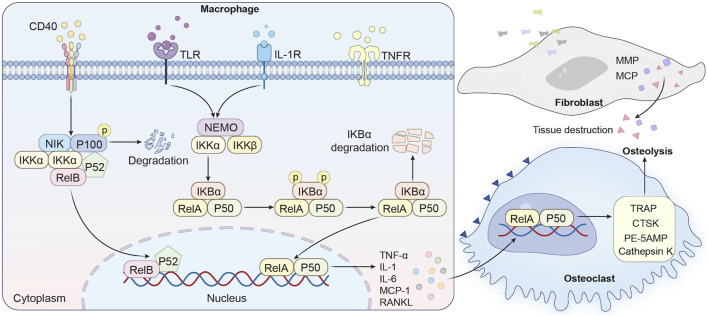
NF-κB activation and therapeutic modulation in periprosthetic osteolysis.

The NF-κB pathway serves as a master regulator of inflammatory bone destruction, playing a pivotal role in the pathogenesis of PPO. In PPO, wear particles such as submicron polyethylene debris and metallic ions act as potent stimuli for NF-κB activation. These particles are phagocytosed by macrophages at the implant-bone interface, engaging Toll-like receptors (TLRs; e.g., TLR4 for polyethylene) or triggering NLRP3 inflammasome assembly. This leads to phosphorylation and degradation of IκBα, enabling NF-κB dimers (e.g., p50/RelA) to translocate into the nucleus and initiate transcription of pro-inflammatory cytokines such as TNF-α, IL-1β, and IL-6 (Xu et al., 2009). These cytokines establish a feedforward loop that amplifies NF-κB activity, enhancing osteoclastogenesis through two mechanisms (Figure 2). First, TNF-α potentiates RANKL sensitivity by binding to TNFR1 on osteoclast precursors, amplifying TRAF6-dependent signaling that converges on NF-κB and NFATc1 activation, a transcriptional master switch for osteoclast differentiation (Peng et al., 2020; Luo et al., 2018). Notably, TNF-α alone can induce osteoclastogenesis *in vitro* under inflammatory conditions, bypassing RANKL requirement in an NF-κB-dependent manner (Li and Zhang, 2021). Second, NF-κB directly induces expression of c-Fos and NFATc1, master regulators of osteoclast-specific genes (e.g., TRAP, CTSK, DC-STAMP) (Chen W. et al., 2019). Genetic deletion of both p50 and p52 in mice leads to osteopetrosis, characterized by impaired osteoclastogenesis, thereby highlighting the indispensable role of the NF-κB pathway in physiological bone resorption. Clinical studies reveal elevated RANKL and NF-κB activity in periprosthetic tissues of patients with implant loosening (Li and Zhang, 2021; Chen W. et al., 2019; Novack, 2011; Zhou et al., 2024). For instance, titanium particles stimulate fibroblasts to secrete MMPs and chemokines (e.g., MCP-1), further recruiting osteoclast precursors and exacerbating bone loss (Jiang et al., 2013; Noordin and Masri, 2012; Landgraeber et al., 2014). Clinical studies consistently demonstrate elevated NF-κB activity in periprosthetic tissues from patients with aseptic loosening (Schwarz et al., 2000; Gao X. R. et al., 2018). Immunohistochemical analyses reveal nuclear localization of p65 in macrophages and fibroblasts adjacent to titanium or cobalt-chrome particles, correlating with increased RANKL/OPG ratios and osteolytic lesion severity (Masui et al., 2005; Noordin and Masri, 2012). Mechanistically, wear particles stimulate fibroblasts to secrete matrix metalloproteinases (MMPs; e.g., MMP-9, MMP-13) and chemokines (e.g., MCP-1), which degrade bone matrix and recruit circulating osteoclast precursors to the implant site (Landgraeber et al., 2014; Hallab and Jacobs, 2017).

### 4.3 Therapeutic modulation of NF-κB signaling

As detailed in the preceding sections, aberrant activation of NF-κB signaling plays a central role in transducing inflammatory stimuli into osteoclast-mediated bone resorption in periprosthetic osteolysis. Given its pivotal involvement in both upstream immune responses and downstream osteoclastogenesis, NF-κB has emerged as a prime therapeutic target. The following section summarizes current strategies to inhibit NF-κB signaling at various molecular levels, aiming to mitigate pathological bone loss while minimizing systemic immunosuppression.

Targeting the NF-κB pathway presents a multifaceted strategy to mitigate PPO. Upstream cytokine neutralization represents a primary therapeutic approach, focusing on TNF-α, a central mediator of wear particle-induced osteolysis. Because of the central role of TNF-α in wear particle-induced osteolysis, several studies have focused on blocking, neutralizing, or silencing the TNF-α ligand in experimental models of osteolysis (Sun et al., 2013; Peng et al., 2016). TNF-α inhibitors (e.g., etanercept) and IL-1 receptor antagonists (e.g., anakinra) suppress NF-κB activation in preclinical models. However, clinical trials show limited efficacy in PPO, likely due to compensatory IL-1 signaling (Yu et al., 2020; Liu et al., 2017). To address this limitation, small interfering RNAs (siRNAs) targeting TNF-α or IL-1β genes have emerged as promising tools for localized silencing of pro-inflammatory mediators, offering precision in modulating NF-κB activity within the bone microenvironment (Cheng and Zhang, 2008). Kinase cascade inhibition provides an alternative strategy to disrupt NF-κB signaling. In addition to traditional IKKβ inhibitors (such as BMS-345541), small molecules targeting upstream kinases such as TAK1 (such as denbinobin) and MEKK3 (such as KSR2 inhibitors) can indirectly inhibit the activation of the IKK complex (Gupta et al., 2010). In addition, the regulation of protein phosphatases (such as WIP1) exerts an inhibitory effect by dephosphorylating the p65 Ser536 site of the NF-κB subunit, providing a new idea for drug development (Roberti et al., 2022). Direct IKK complex inhibition has been extensively explored. Small-molecule inhibitors (e.g., BMS-345541) block IKKβ activity, reducing IκB degradation and NF-κB nuclear translocation. Peptide inhibitors targeting NEMO-IKK interactions also demonstrate anti-osteolytic effects *in vivo* (Di Francesco et al., 2022). Recent mechanistic studies highlight the role of post-translational modifications in regulating NF-κB activity. For instance, ISGylation of NEMO at lysine 270 (K270) stabilizes autophagosomes, enhancing IκBα degradation and increasing p65 nuclear translocation efficiency by 3.2-fold (Adapala et al., 2020). Similarly, tetrandrine binds directly to the IKKβ catalytic domain (binding energy: −10.3 kcal/mol), suppressing IκBα phosphorylation by 78% (Liu et al., 2020). However, compensatory activation of IKKα following IKKβ inhibition necessitates combinatorial therapies with subunit-specific inhibitors to overcome drug resistance (Huynh et al., 2000). Proteasome inhibitors, such as bortezomib, stabilize IκBα by preventing its proteasomal degradation, thereby attenuating NF-κB activation. Preclinical studies in titanium particle-induced osteolysis models validate their bone-protective effects (Van Stiphout et al., 2022). Innovative approaches include bioactive compounds like chalcone A, which suppressed osteoclastogenesis by inhibiting IκBα phosphorylation (67% reduction) and p65 nuclear translocation (54.3% decrease, P < 0.001) in murine models (Liao et al., 2021). Gene therapy strategies aim to achieve localized NF-κB suppression. Adenoviral delivery of dominant-negative IKKβ (IKKβdn) or super-repressor IκBα (IκBαSR) effectively inhibits NF-κB in target tissues. Despite promising preclinical results, challenges such as viral vector immunogenicity and off-target effects persist. To circumvent these limitations, nanoparticle-encapsulated TNF-α/IL-1β siRNA systems have been developed, demonstrating macrophage-targeted delivery and enhanced efficacy in regulating the bone microenvironment (Zapata Lopera et al., 2022). For example, targeted knockout of p65 reduced osteolytic metastasis formation in breast cancer by 82%, as quantified by micro-CT (Li et al., 2021). Future directions emphasize the integration of single-cell sequencing and organoid models to dissect NF-κB signaling heterogeneity within the bone microenvironment. Such advances will enable the design of precision interventions tailored to individual patient profiles, bridging the gap between experimental models and clinical translation.

## 5 MAPK/ERK signaling pathway

The MAPK/ERK signaling cascade precisely regulates osteoclast differentiation and function by integrating mechanical and biochemical signals. To elucidate its multidimensional mechanisms of action, the following sections will systematically describe the structural composition of this pathway and its dynamic regulation in bone metabolism.

### 5.1 Structural composition of MAPK/ERK signaling

The MAPK family, comprising serine/threonine-specific kinases, plays a central role in regulating cellular processes such as proliferation, differentiation, apoptosis, and stress responses (Guo et al., 2020). In mammals, the MAPK family is categorized into three major subfamilies: Extracellular Signal-Regulated Kinases (ERK1/2), p38 kinases (p38α, p38β, p38γ, p38δ), and c-Jun N-terminal Kinases (JNK1/2/3). ERK1/2 is primarily activated by growth factors and mitogens, governing cell survival and differentiation through phosphorylation of downstream substrates such as transcription factors (e.g., c-Fos, CREB) and cytoskeletal proteins. p38 kinases, in contrast, respond to inflammatory cytokines (e.g., TNF-α, IL-1β) and environmental stressors, mediating inflammatory signaling and apoptosis. JNKs are activated by oxidative stress and genotoxic agents, modulating stress-induced apoptosis and inflammatory cascades (Canovas and Nebreda, 2021; Lucas et al., 2022). The MAPK signaling cascade operates through a hierarchical three-tiered kinase module. First, MAP kinase kinase kinases (MAP3Ks; e.g., Raf, MEKK1, apoptosis signal-regulating kinase 1 [ASK1]) are activated by upstream signals such as Ras GTPases or cytokine receptors. Second, MAP kinase kinases (MAP2Ks; e.g., MEK1/2 for ERK, MKK3/6 for p38, MKK4/7 for JNK) phosphorylate and activate the terminal MAPKs. Third, the activated MAPKs (ERK, p38, or JNK) phosphorylate downstream targets, including transcription factors and regulatory proteins, to execute context-specific cellular responses (Keshet and Seger, 2010). Of these subfamilies, ERK1/2 is the most extensively studied effector kinase. It functions within a highly specific three-layered cascade, playing pivotal roles in cell cycle progression, survival, and differentiation. For example, ERK activation by growth factor signaling promotes cell proliferation via cyclin D1 upregulation, while its sustained activity in stressed cells may paradoxically drive apoptosis or senescence, depending on cellular context.

### 5.2 Role of MAPK/ERK signaling and bone resorption

**FIGURE 3:**
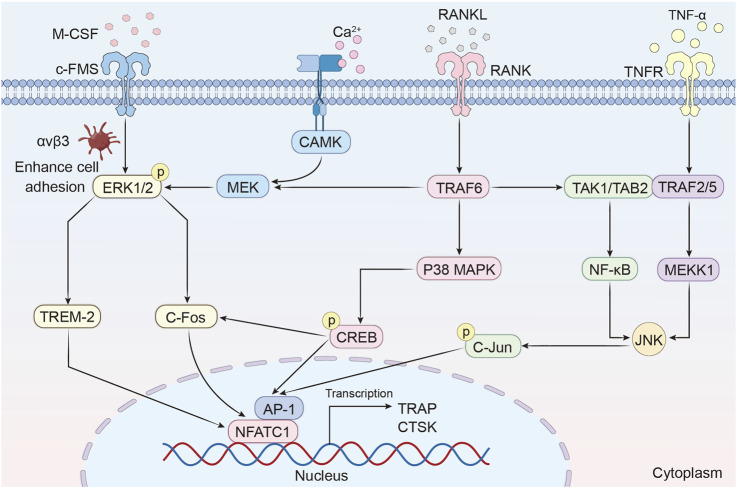
MAPK/ERK signaling regulates osteoclast differentiation and bone resorption.

MAPK pathways are pivotal in osteoclast differentiation and bone resorption, orchestrating cellular responses through distinct yet interconnected mechanisms (Figure 3).

#### 5.2.1 ERK1/2 signaling: sustained activation drives osteoclastogenesis

The ERK signaling pathway has been implicated in the survival, proliferation, apoptosis, formation, polarity, podosome disassembly, and differentiation of osteoclasts. Sustained activation of extracellular signal-regulated kinase 1/2 (ERK1/2) is critical for osteoclast survival and differentiation. The synergistic action of Macrophage colony-stimulating factor (M-CSF) and RANKL orchestrates osteoclastogenesis through a coordinated receptor-signaling axis. Mechanistically, M-CSF binding to c-Fms activates integrin αvβ3 to enhance cell adhesion, which synergizes with RANKL-RANK signaling to potentiate ERK1/2 phosphorylation and osteoclast differentiation (Hodge et al., 2011; Ross and Teitelbaum, 2005). The activation of ERK1/2 directly induces the expression of c-Fos, which is an upstream transcription factor of NFATc1 (Feng and Teitelbaum, 2013). Notably, ERK1 exerts a more pronounced impact on osteoclast differentiation than ERK2, as evidenced by reduced osteoclast formation and increased bone density in ERK1 knockout mice (He et al., 2011). IL-1α further enhances osteoclast survival by inhibiting apoptosis through ERK-dependent pathways (Lee et al., 2002). Additionally, the calmodulin-dependent kinase (CaMK)-MEK-ERK axis amplifies NFATc1 expression via upregulation of triggering receptor expressed on myeloid cells-2 (TREM-2), establishing a feedforward loop that reinforces osteoclast differentiation (Park-Min et al., 2009).

#### 5.2.2 p38 MAPK-CREB axis: dual roles in osteoclast regulation

p38 MAPK regulates osteoclast differentiation through a dual mechanism. On one hand, RANKL stimulates p38 MAPK to phosphorylate cAMP response element-binding protein (CREB), which upregulates c-Fos and NFATc1 to drive osteoclast-specific gene transcription. On the other hand, the scaffolding protein B-cell adapter for phosphoinositide 3-kinase (BCAP) amplifies this signaling by enhancing CREB activation, while ameloblastin (Ambn) suppresses osteoclastogenesis by competitively inhibiting p38-CREB interactions (Koga et al., 2019; Chaweewannakorn et al., 2019). A vivo study demonstrates that mice lacking p38α exhibit increased bone mass and reduced resorption, whereas the specific p38 inhibitor SB203580 blocks precursor differentiation without affecting mature osteoclast function (Cong et al., 2017).

#### 5.2.3 JNK pathway: synergy with NF-κB in osteoclast activation

The c-Jun N-terminal kinase (JNK) pathway is activated by TNF-α or RANKL, inducing activator protein-1 (AP-1) transcription factor activity. JNK synergizes with NF-κB to drive the expression of osteoclast-specific genes such as tartrate-resistant acid phosphatase (TRAP) and cathepsin K (CTSK) (Park et al., 2017). Among JNK isoforms, JNK1 plays a dominant role in osteoclast differentiation, as Jnk1 knockout mice exhibit diminished AP-1 activity and impaired NFATc1 induction (Kim and Kim, 2016). Pharmacological inhibition of JNK with SP600125 dose-dependently suppresses osteoclast formation, highlighting its therapeutic potential (Lee et al., 2018; Jang et al., 2023).

#### 5.2.4 Crosstalk between MAPK subfamilies in osteoclast metabolism

MAPK signaling pathways, including ERK, JNK, and p38 MAPK, play pivotal roles in the regulation of osteoclast metabolism. These kinases are not only activated by distinct upstream stimuli—such as M-CSF for ERK and RANKL or pro-inflammatory cytokines for JNK and p38—but also engage in intricate crosstalk that orchestrates osteoclast proliferation, differentiation, fusion, and bone-resorptive function (Lee et al., 2018; Lee et al., 2016). ERK primarily supports osteoclast precursor proliferation and early differentiation by inducing transcription factors such as c-Fos and NFATc1. In contrast, JNK and p38 are more involved in promoting osteoclast maturation and fusion through the activation of AP-1 components and NFATc1 phosphorylation, as well as reorganization of the actin cytoskeleton (Lee et al., 2018; Kim et al., 2020).

Recent evidence highlights that MAPK subfamilies dynamically regulate each other via feedback and feedforward mechanisms. For instance, ERK activation enhances the expression of dual-specificity phosphatases (DUSPs), which negatively regulate JNK activity, while inhibition of ERK can release this suppression, leading to compensatory activation of p38 and JNK pathways (Fey et al., 2012). Moreover, p38 signaling has been shown to attenuate ERK activity via phosphatase recruitment, promoting the transition from precursor proliferation to terminal osteoclast differentiation (Zhang et al., 2014). This inter-pathway modulation ensures tight control of signaling intensity and duration, which is essential for appropriate cell fate decisions. Transient ERK or JNK activation tends to support survival and proliferation, whereas sustained activation may lead to apoptosis or terminal differentiation depending on the cellular context (Fey et al., 2012; Pearson et al., 2001).

Functionally, the crosstalk between MAPK pathways is critical for osteoclast fusion, cytoskeletal dynamics, and bone-resorbing capacity. JNK and p38 signaling jointly regulate the expression of fusion-related genes such as DC-STAMP and ATP6V0D2, and their simultaneous inhibition results in impaired osteoclast multinucleation and resorption activity (Kim et al., 2020). Furthermore, during pathological bone loss such as in inflammatory osteolysis, the exaggerated activation of JNK and p38 contributes to excessive osteoclastogenesis, whereas ERK-mediated negative feedback mechanisms attempt to limit this response (Sharma et al., 2020; Lee et al., 2018). Notably, pharmacological inhibition of individual MAPK pathways using agents like U0126 or SB203580 has revealed compensatory activation of the others, underscoring the therapeutic challenge posed by the redundancy and interdependence within the MAPK network (Henklova et al., 2008; You et al., 2022). Thus, understanding the precise mechanisms of MAPK crosstalk in osteoclasts may offer novel strategies for the development of combination therapies aimed at modulating bone resorption more effectively.

### 5.3 Regulation of MAPK/ERK signaling: therapeutic strategies and mechanobiological insights

In addition to NF-κB, the MAPK/ERK signaling cascade constitutes another critical axis mediating osteoclast differentiation and activity in response to inflammatory and mechanical cues. Sustained activation of MAPK pathways, particularly ERK and p38, amplifies osteoclastogenic transcription programs, reinforcing the progression of periprosthetic bone loss. This section discusses pharmacological and mechanobiological strategies that target MAPK/ERK signaling to restore bone homeostasis and prevent implant failure.

The MAPK/ERK signaling cascade, a pivotal regulator of osteoclast differentiation and bone resorption, has emerged as a promising therapeutic target for bone metabolic disorders. Current strategies to modulate this pathway span pharmacological, genetic, and mechanobiological approaches, each with distinct mechanisms and clinical implications. Pharmacological interventions focus on disrupting key signaling nodes, with first-line agents such as alendronate and zoledronate exerting anti-resorptive effects through inhibition of the mevalonate pathway. By preventing post-translational prenylation of small GTPases (e.g., RhoA, Rac1), bisphosphonates impair membrane localization of these proteins, thereby suppressing downstream ERK and PI3K/Akt signaling essential for osteoclast survival and F-actin ring formation (Gong et al., 2011; Tsubaki et al., 2014). Clinical trials demonstrate that bisphosphonates reduce periprosthetic osteolysis risk by 30%–40% in total hip arthroplasty patients, though concerns persist regarding atypical femoral fractures with long-term use (Di Martino et al., 2024; Khan and Kaiser, 2017). Natural compounds offer complementary therapeutic potential. For instance, acetyl-11-keto-β-boswellic acid (AKBA), a bioactive triterpene derived from Boswellia serrata, selectively inhibits ERK1/2 phosphorylation at Thr202/Tyr204 via direct binding to c-Raf kinase. At 10 μM, AKBA reduces osteoclast size by 60% and resorption pit area by 75% *in vitro* by destabilizing the F-actin sealing zone (Shi et al., 2021). Synergistic effects are observed when AKBA is combined with RANKL-neutralizing antibodies, suggesting utility in combinatorial therapies. Similarly, the flavonoid baicalin exhibits dose-dependent bidirectional modulation of osteoclastogenesis, as demonstrated by Lu et al. (2018) through integrated *in vitro* and *in vivo* models. Pharmacological inhibition with U0126 confirmed ERK pathway mediation of these effects, positioning baicalin as a novel dual-modulator of bone remodeling with dosage-dependent therapeutic implications (Lu et al., 2018). Genetic and epigenetic modulation strategies further expand the therapeutic arsenal. Knockout of MAPK14 (encoding p38α) in osteoclast precursors reduces bone resorption and increases bone mass in murine models (Lee et al., 2018). SiRNA-mediated silencing of mitogen-activated protein kinase kinase 6 (MKK6) attenuates RANKL-induced osteoclast differentiation by disrupting upstream MAPK activation (Huang et al., 2006; Matsumoto et al., 2000). Mechanobiological insights highlight the role of mechanical stress in regulating MAPK/ERK signaling. Mechanical unloading upregulates RANKL expression and enhances MAPK activity, linking disuse osteoporosis to accelerated osteoclast-mediated bone loss (Wang L. et al., 2022; Wang et al., 2020; Liu et al., 2022). These findings underscore the importance of biomechanical cues in maintaining bone homeostasis and suggest adjunctive strategies such as physical loading regimens to counteract pathological resorption.

## 6 Conclusion

Osteoclast differentiation and bone resorption are tightly regulated by a network of signaling pathways, with imbalances in these processes serving as the core mechanism underlying PPO and other bone metabolic disorders. This review comprehensively delineates the roles of the RANKL/RANK/OPG, NF-κB, and MAPK/ERK pathways in osteoclastogenesis and pathological bone loss. The RANKL/RANK/OPG axis acts as the master regulator of osteoclast differentiation, where RANKL-RANK binding initiates downstream signaling cascades, while OPG functions as a critical decoy receptor to maintain bone homeostasis. Recent advances have elucidated the spatiotemporal dynamics of RANKL expression and its crosstalk with immune cells, providing novel insights into bone-immune interactions (Ono et al., 2020). The NF-κB pathway, particularly its canonical branch, serves as a central hub integrating inflammatory signals (e.g., TNF-α, IL-1β) with osteoclast activation. Both genetic and pharmacological inhibition of NF-κB demonstrate significant anti-osteolytic effects, yet challenges remain in achieving tissue-specific targeting to avoid systemic immunosuppression. The MAPK/ERK pathway further amplifies osteoclast activity through CREB-mediated transcriptional activation of c-Fos and NFATc1, highlighting its synergistic role with RANKL and NF-κB signaling.

### 6.1 Clinical translation challenges

Emerging therapeutic strategies, including monoclonal antibodies, small-molecule inhibitors, and natural compounds, hold promise for mitigating PPO. However, The therapeutic strategies targeting key signaling pathways (RANKL/RANK/OPG, NF-κB, and MAPK/ERK) discussed in Section 3, Section 4, Section 5 face significant clinical translation barriers that necessitate critical evaluation. Small-molecule inhibitors effectively suppress NF-κB-driven osteoclastogenesis in preclinical models but risk systemic immunosuppression and hepatotoxicity. For example, IKKβ inhibitors like BMS-345541 have shown efficacy in preclinical models, but their clinical application is complicated by significant risks, including systemic immunosuppression and hepatotoxicity (Zhang et al., 2023). These side effects can compromise the patient’s overall health, making long-term treatment with these inhibitors problematic for managing PPO. Chronic pharmacological inhibition of IKKβ compromises innate immunity by persistently suppressing NF-κB signaling. This sustained blockade impairs the pathogen clearance capacity of macrophages and neutrophils, increasing infection susceptibility. It also disrupts essential pro-inflammatory cytokine feedback loops (e.g., TNF-α, IL-1β), mirroring the immunodeficiency phenotypes observed in genetic models of NF-κB deficiency, such as the p50/p52 double-knockout mice (Wakamatsu et al., 2005; Gupta et al., 2010; Ruocco et al., 2005). Biologics, such as Denosumab (anti-RANKL mAb), demonstrate potent osteoclast inhibition but incur significant clinical trade-offs. These include a higher incidence of hypocalcemia and rebound resorption post-cessation due to RANKL accumulation, as well as limited implant-site bioavailability that necessitates high systemic doses (Kumar et al., 2024; Fu et al., 2023). Local delivery of genetic payloads (e.g., siRNA or CRISPR constructs) can selectively inhibit key pathways, but efficacy is constrained by sub-40% macrophage transfection rates and prevalent anti-adenovirus immunity in up to 60% of recipients (Davis et al., 2010). Advances in nanoparticle carriers (e.g., PEGylated liposomes) show promise for enhancing targeting and reducing immunogenicity, though *in vivo* validation remains limited. Natural compounds, such as AKBA, offer multi-pathway modulation in PPO therapy. However, their poor pharmacokinetics, including an oral bioavailability of less than 5% due to first-pass metabolism, significantly limit their therapeutic potential. To overcome this, modifications such as bisphosphonate conjugation for targeted bone delivery are necessary (Farrell et al., 2018; Krüger et al., 2008).

These limitations underscore why single-target approaches show suboptimal clinical outcomes in PPO. As emphasized in Section 2.1, Section 2.2, the inflammatory cascade in periprosthetic tissues involves redundant cytokine networks (TNF-α/IL-1β/IL-6 crosstalk) and cellular heterogeneity (M1/M2 macrophage dynamics), demanding combinatorial strategies that concurrently address multiple pathological axes.

### 6.2 Future perspectives

Current approaches often target single pathways, overlooking the complexity of inter-pathway crosstalk. For instance, compensatory activation of IL-1 signaling upon TNF-α inhibition underscores the need for multi-target interventions. To overcome these limitations, multi-target approaches show superior potential: Bispecific antibodies (e.g., RANKL/IL-6 dual-targeting mAbs) concurrently disrupt inflammatory amplification and osteoclast differentiation pathways. The anti-TNF/IL-6 bispecific antibody completely inhibits the inflammatory factor CXCL13 in the arthritis model, and its effect is superior to that of the monoclonal antibody combination (Biesemann et al., 2023). A further evaluation of the anti-TNF/IL-6 antibodies’efficacy in clinical studies is required; Kinase cascade co-inhibition (e.g., TAK1 + ERK inhibitors) synergistically suppresses MAPK/NF-κB crosstalk. TAK1 inhibitors significantly inhibit the activation of NFATc1 by suppressing NF-κB and part of the MAPK pathway, while ERK inhibitors block the direct regulation of NFATc1 by the MAPK pathway. The two work together to block the cross-dialogue between MAPK and NF-κB (especially the positive feedback loop of TAK1-ERK-NF-κB) (Li L. et al., 2015; Li M. et al., 2015). In an ovalbumin (OVA)-induced asthma mouse model, dual inhibition of NF-κB and p38 MAPK reduced airway inflammation (Kim et al., 2011). Future research should prioritize multi-omics integration to dissect signaling heterogeneity in the periprosthetic microenvironment. Single-cell RNA sequencing and spatial transcriptomics could map dynamic interactions between osteoclast precursors, immune cells, and stromal components, identifying nodal points for therapeutic intervention. Concurrently, Advanced drug delivery systems, such as nanoparticle-based carriers engineered for localized and sustained release of pathway-specific inhibitors, may enhance therapeutic precision while minimizing systemic toxicity. Mechanobiological insights into how mechanical stress reprograms osteoclast activity via pathways like Piezo1/Yes-associated protein (YAP) signaling could inform adjunctive biomechanical therapies. For instance, optimizing implant design or postoperative loading protocols might counteract pathological bone remodeling (Wang et al., 2020). Emerging biomaterial strategies, such as copper (Cu)-based implant coatings, offer multi-modal protection against PPO. Cu ions suppress wear particle-induced inflammation by inhibiting macrophage TLR/NF-κB signaling and TNF-α/IL-1β release, thereby reducing osteoclast activation (Batool et al., 2021). Additionally, it promotes macrophage polarization toward the M2 phenotype, fostering an anti-inflammatory microenvironment (Wang D. et al., 2022). Furthermore, its antibacterial properties and ability to enhance osteointegration contribute to reduced infection and lower generation of wear particles (Wang et al., 2017).

In conclusion, while significant progress has been made in understanding the molecular basis of PPO, translating these findings into clinical practice requires a paradigm shift toward combinatorial therapies and precision medicine. Bridging the gap between experimental models and human pathophysiology will be essential to address unmet needs in bone metabolic disease management.
